# Flavor and Texture Characteristics of ‘Fuji’ and Related Apple (*Malus domestica* L.) Cultivars, Focusing on the Rich Watercore

**DOI:** 10.3390/molecules25051114

**Published:** 2020-03-02

**Authors:** Fukuyo Tanaka, Fumiyo Hayakawa, Miho Tatsuki

**Affiliations:** 1 Central Region Agricultural Research Center, National Agriculture and Food Research Organization (NARO), Tsukuba 305-8666, Japan; 2 Food Research Institute, National Agriculture and Food Research Organization (NARO), Tsukuba 305-8642, Japan; 3 Institute of Fruit Tree and Tea Science, National Agriculture and Food Research Organization (NARO), Tsukuba 305-8605, Japan

**Keywords:** ‘Fuji’, watercore, sweetness, flavor, texture, flesh browning disorder, apple

## Abstract

Watercore is a so-called physiological disorder of apple (*Malus domestica* L.) that commonly occurs in several well-known cultivars. It is associated with a rapid softening of the flesh that causes a marked changed in flavor and texture. In Asia, apples with watercore are preferred and considered a delicacy because of their enhanced sweet flavor. The ‘Fuji’ cultivar, the first cultivar with rich watercore that is free from texture deterioration, has played a key role in the development of the market for desirable watercored apples. This review aimed to summarize and highlight recent studies related to the physiology of watercore in apples with special focus on ‘Fuji’ and related cultivars.

## 1. Introduction

The ‘Fuji’ cultivar has maintained a large share of the global apple production market over the last two decades [1]. Originally, ‘Fuji’ was selected from cross between ‘Ralls Janet’ and ‘Delicious’ in the Tohoku region of Japan and gained popularity because it is extremely juicy and crisp with a sweet flavor similar to that of ‘Delicious’ [2]. ‘Fuji’ is also susceptible to watercore development. Watercore is a phenomenon that presents as a translucent appearance at the core and/or flesh of the fruit, and it is caused by the intercellular spaces of the affected tissue being filled with fluid. It has been reported that watercore development is related to sugar metabolism during the maturation process and fruit mineral composition. Watercored apple is prone to several physiological disorders, such as browning and breakdown during storage [3,4,5,6,7,8,9]. Furthermore, strains of the ‘Delicious’ cultivar are also highly susceptible to watercore, which is typically accompanied by changes in texture traits, such as softening and mealiness. These undesirable characteristics that commonly occur in watercored ‘Delicious’ strains have caused watercoring in apples to be viewed negatively.

In spite of this, watercored ‘Fuji’ has gradually become desirable in Japan and other Asian countries, and the palatability of ‘Fuji’ and watercored apples has been identified in the last decade. Today, Japanese producers and consumers generally value watercored apple owing to its excellent fruit flavor, which occurs when it fully matures on the tree. In fact, watercored apples are often advertised using phrases such as aroma-rich and pineapple-like. Furthermore, the rich watercore trait has become a breeding target with the aim of increasing the sweet flavor in apple [10]. Aprea et al. [11] proposed that apple breeding programs must take into account factors such as volatile compounds, texture parameters, minor components, and information from sensory panels. However, perceived sweetness is difficult to be described because it is always perceived in combination with other sensory properties, which influence its evaluation. Sweetness perception is a complex and multisensory process, and only gustatory stimuli are insufficient to fully understand and predict it [12]. In this work, we focus on ‘Fuji’ and the related cultivars and review the mechanism of watercoring in apple palatability. We also assess various characters, such as flavor, texture, and genetic properties, employing integrated analysis of instrumental and sensory profiling.

## 2. Flavor Characteristics

### 2.1. Sensory Analysis

Although watercored apple has been extremely popular among Japanese consumers, there were little published data regarding the overall acceptance for watercored apple available. In order to characterize the unique flavor and overall acceptance, Tanaka et al. [13] conducted sensory analysis using watercored and nonwatercored ‘Fuji’ with 29 trained panelists. With respect to overall acceptance, watercored apples scored significantly higher than nonwatercored apples (Table 1). Overall intensities of aroma and taste and five sensory attributes were scored using a seven-point scale. Taste intensity was evaluated with nose clip. Overall aroma intensity and perception of sweet and fruity flavors were enhanced in watercored apple, whereas green and sour perception was enhanced in nonwatercored apple (Table 1). Overall taste intensity, in which the influence of aroma was eliminated by clips, was not significantly different, indicating that the contribution of aroma to the overall acceptance and characteristics of flavor was remarkably large in this case.

### 2.2. Analysis of Volatile and Water-Soluble Compounds

**Figure 1:**
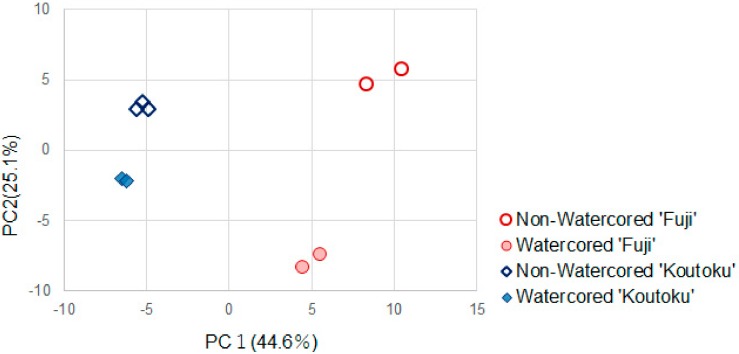
Principal component analysis score plots of the volatiles and solubles of fruit juice. PC1 and PC2 scores were discriminated by cultivars and watercore existence, respectively. Top 10 of PC2 loadings were (1) ethyl butanoate, (2) ethyl propanoate, (3) ethyl 2-methylbutanoate, (4) ethyl acetate, (5) ethyl hexanoate, (6) sorbitol, (7) galactaric acid, (8) methyl 2-methylbutanoate, (9) methyl acetate, and (10) ethyl tiglate. Reproduced with permission from Tanaka et al. [13].

Volatile and water-soluble components were profiled for watercored and nonwatercored ‘Fuji’ and ’Koutoku’, a progeny of ’Fuji’ (Tables S1 and S2) [13]. In both cultivars, ethyl esters and methyl esters of fatty acids were detected in watercored fruit; their peak intensities were as large as several to several hundred times those of nonwatercored apple (Table S1). In addition, principal component analysis of the intensities of the 109 components suggested that the PC1 score was differentiated by cultivar, whereas the PC2 score was differentiated by the presence of watercore (Figure 1). The PC2 loadings suggested that ethyl esters, methyl esters, sorbitol, galactaric acid, erythronic acid, and dehydroascorbic acid were associated with watercore. Increase in sorbitol was consistent with previous reports [14,15,16,17]. This integrated profiling analysis suggested that an increase in methyl esters and ethyl esters is crucial to the attributes and desirability of watercored apple. Similar phenomena have been revealed by Dixon et al. [18] when, following a short-term exposure to hypoxic conditions, time courses of apple aroma components and odor units for 10 apple cultivars were analyzed, and their results indicated that odor unit values highly corresponded to ethyl ester levels.

**Figure 2:**
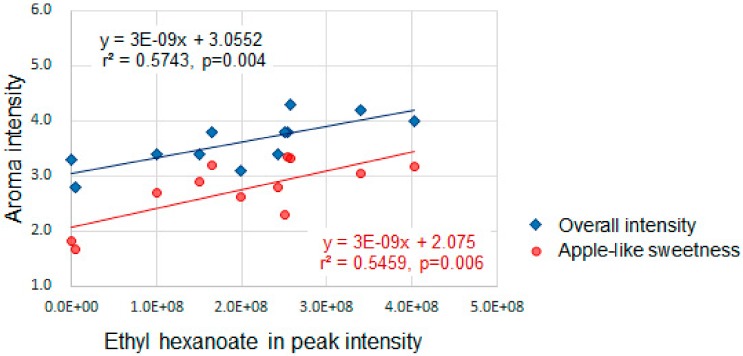
Ethyl hexanoate and aroma intensity in ester-enhanced ‘Fuji’ by incubation with ethanol mixture. Ethyl hexanoate is shown as a representative of ethyl esters because major ethyl esters of ‘Fuji’ correlate with one another in their peak intensities.

Ethyl esters have been reported to have an apple-like, fruity, sweet aroma with an extremely low threshold value. For example, Komthong et al. [19] analyzed head-space volatiles of ‘Fuji’ using aroma extract dilution analysis and determined flavor dilution factor, which is the lowest dilution ratio of the volatile compounds. Then, methyl 2-methylbutanoate and ethyl 2-methylbutanoate were estimated and determined to be the most potent odorants in the volatiles based on their lowest threshold odor values. Moreover, we demonstrated that an increase in ethyl esters significantly enhanced the perception of apple-like sweetness by sensory evaluation using a series of ‘Fuji’ samples that had chemically modified aroma (Figure 2). Based on these data, ethyl esters appear to be potent, key flavor compounds in watercored apples.

The difference among sugar and sorbitol contents, soluble solid contents (SSC), and gene expression related to sugars in watercored and nonwatercored tissues has been studied extensively [14,16,20,21]. Specifically, sorbitol accumulation in the watercored tissues was observed in several cases, whereas fructose, glucose, sucrose, total sugar contents, and SSC were only observed in a few cases [13,15,16,20]. Fructose, the sweetest sugar of the apple components [22,23], tended to be lower in watercored tissues. Sorbitol tends to be present at a low content and exhibits weaker perceptive sweetness compared with other sugars, even though it increases in content in watercored tissues. [23,24]. The comparison between watercored and nonwatercored tissues often found decreases in sweetness in the watercored tissues. According to Melad-Herreros [15] and Williams et al. [16], nonaffected tissues of watercored apples, often edible parts, scored higher for fructose and sucrose than the affected tissues (Table 2), indicating that watercored apples may in fact be sweeter. Harker et al. [25] stated that two apples needed to differ in °Brix (SSC) by more than 1 before evoking a change in response to a perceived sweet taste for the median panelist. A difference of 1 °Brix corresponds to 1% difference in sucrose. Table 2 presents the estimated sweetness using two equations that defined sucrose sweetness as 1 [26,27]. Our estimation (a) took sorbitol into account based on the estimation summarized by Kitahata and Machinami [22], whereas (b), known as the total sweetness index, did not [23]. Williams et al. [17] also found that the difference between nonwatercored and nonaffected tissues of watercored apples was nearly 1. In this case, there was a perceived sweetness difference between the edible part of a watercored and that of a nonwatercored apple at a near-threshold level. These findings are in agreement with the results of sensory evaluation (Table 1) of taste intensity, which found that while watercored apples were generally evaluated a little intense, they did not differ significantly from control samples. Given these findings, the significant difference in sweetness is likely affected by components other than sugars.

Recently, the importance of aroma components in the characteristics of flavor and preference in apple has been widely recognized. Aprea et al. [11] reported that sorbitol content correlated with perceived sweetness better than any other single sugar or total sugar content. Furthermore, their predictive model based on partial least squares regression included not only SSC but also volatile compounds and revealed that several volatiles are possibly contributing to flavor. Having a sweet taste is an important but difficult attribute to be predicted using objective measurements [25]. The contribution of sugars to the enhancement of perceived sweetness in watercored apple is likely limited, whereas the profile of aroma components varies widely and accounts for several of the unique flavor profiles. Aroma components between watercored and nonwatercored apples can be markedly different. For instance, our analysis revealed that the detected levels of most ethyl esters that created an aroma profile with characteristics similar to pineapple or ginjoshu (high-quality sake) were ten times their levels in nonwatercored apples (Table S1) [13,28,29,30,31]. Considering these profiles of flavor components and sensory attributes, the contribution of aroma components, such as ethyl esters, is crucial in producing the flavor characteristics in watercore-rich apples.

### 2.3. Mechanism of Enhanced Ethyl Ester Synthesis in Watercored Apples

**Figure 3:**
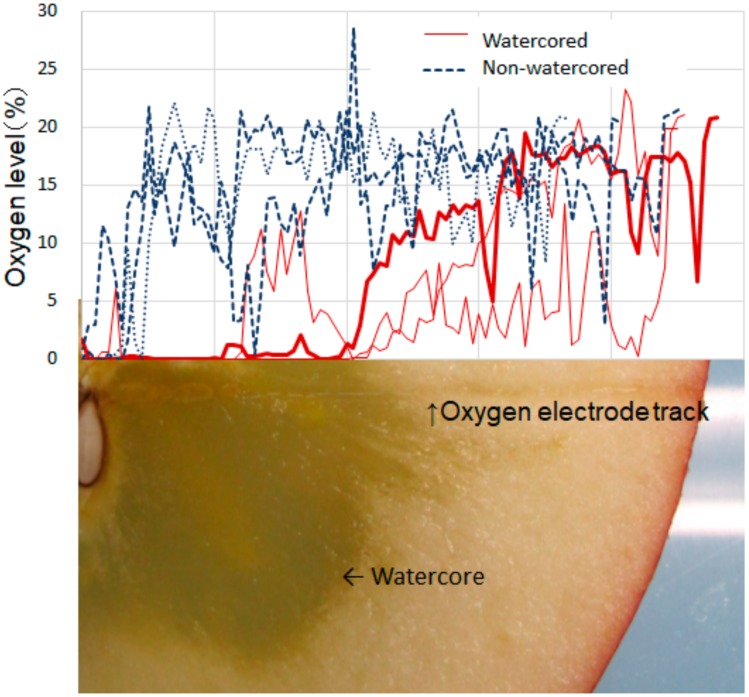
Oxygen distribution related to watercore of ‘Fuji’ apple. Three fruits were measured for each class. Thick red line corresponds to the photograph. Reproduced with permission from Tanaka et al. [13].

Because ethyl esters are crucial in aroma and flavor profiles in apples, different analyses have already focused on the synthesis. Dixon and Hewett [18] reported that apple volatile compounds increased in ethanol and ethyl ester concentrations after exposure to hypoxic conditions. Specifically, the synthesis of ethyl esters was high in watercore-susceptible cultivars ‘Red Delicious’, synonymous with ‘Delicious’, and ‘Fuji’ and low in nonsusceptible cultivars ’Golden Delicious’ and ’Cox’s Orange Pippin’. It has also been reported that ethyl esters from apples subjected to controlled-atmosphere (CA) storage exhibited a temporary increase in ethanol and ethyl ester concentrations. Hypoxia likely activates anaerobic glycolysis and ethanol synthesis, causing an increase in ethyl ester production [32,33]. One study found that a decrease in respiration and an increase in ethanol and acetaldehyde concentrations in watercored tissues of ’Richard Delicious’, a sport of ‘Delicious’, shared similarity with apples that were exposed to hypoxic conditions or CA-stored [3]. Furthermore, Tanaka et al. [13] analyzed oxygen distribution within a fruit and demonstrated low-oxygen status at the watercored position (Figure 3), whereas nonwatercored fruits were flat. These phenomena support the concept that ethyl ester synthesis is enhanced under hypoxic conditions within watercored tissues, resulting in distinctive, fermented flavor.

Questions also arise regarding discrimination of volatiles and related gene expression in a fruit associated with oxygen levels. This highly variable, cultivar-dependent response of apple cultivars to hypoxic conditions may also be associated with the physiological processes involved in the development of watercore, which has not been fully elucidated to date. To better understand the metabolism of the characteristic aroma profiles, a fusion analysis of molecular biology and metabolomics will be required.

## 3. Texture Characteristics

### 3.1. Apple Cultivars and Texture Measurement

Texture is a key factor that affects consumer preference of apple [34,35,36]. Texture comprises crispness, mealiness, juiciness, firmness, and other traits and has been reported to influence perceived sweetness [25,37]. Among the texture traits, crispness and juiciness are favorable for apple, whereas softness or mealiness are avoided [35]. Traditional watercore-susceptible cultivars were often accompanied by mealiness and rapid softening [5]. However, the occurrence of watercore and softening is under separate regulations, and cultivars have been developed with one but not the other [10,38]. Here, we review the studies on apple texture as it is related to watercore susceptibility.

Crispness is a sensory and integrated attribute defined as the amount and pitch of sound generated when the fruit is first bitten using the incisor [37,39,40], and it is often estimated from firmness because the two traits are highly and positively correlated [35]. Softening is usually caused by a reduction in firmness, which is typically measured using a penetrometer or sensory analysis. Cell shape, cell size, cell packing, and overall fruit anatomy as well as chemistry of the cell wall and membrane and the role of cell turgor affect firmness [41]. Among them, macromolecular network structures of cell walls, mainly comprising pectin, hemicellulose and cellulose, confer flesh cell rigidity, however, the structures are gradually lost by the cell-wall-modifying enzymes such as β-galactosidase, α-L-arabinofuranosidase, polygalacturonase, pectin methylesterase, and others. Ethylene reportedly stimulated these enzymes, subsequently causing flesh softening. Turgor reduction was also associated with firmness reduction [41,42]. Although cell membranes of apple are not typically associated with cell wall swelling and juiciness [41], so far as watercore is concerned, it may play roles in apple juiciness to some extent, as described below (Section 3.2).

Mealiness is defined as the amount of small, lumpy particles that become apparent during chewing in sensory analysis [37,39,43]. It is due to the loss of cell–cell adhesion or cell separation [37]. Iwanami et al. [38] investigated 23 cultivars and a breeding line under a time-course experiment to evaluate firmness and mealiness and divided them into four groups based on their results after 40 days of storage at 20 °C. The watercore-susceptible cultivars ‘Starking Delicious’ and ‘Red Gold’ were placed in the most rapid mealiness developing group, whereas ‘Fuji’ was the firmest and most nonmealy cultivar. This was consistent with other previous studies [44,45,46]. Iwanami et al. [47] also found that the softening performance of an apple cultivar during storage was highly dependent on the degree of mealiness and turgor reduction rate. The softening rates of all mealy cultivars were high; moreover, the softening rates of nonmealy cultivars were significantly correlated with the turgor reduction rates. In other words, nonmealy cultivars with slow turgor reduction can be expected to exhibit high storage performance. ‘Fuji’ had the lowest turgor reduction rate, which most likely contributed to its firmness and crispness. In addition, ‘Starking Delicious’, another sport of ‘Delicious’, surprisingly exhibited the slowest turgor reduction rate among the tested cultivars contrary to its trait of rapid softening. ‘Fuji’ seems to inherit the excellent trait of slow turgor reduction from the softening cultivar ‘Delicious’ and not from the slow softening ‘Ralls Janet’. Differences in storage performance between ‘Fuji’ and the other ‘Delicious’ strains may mainly be due to differences in mealiness or nonmealiness.

A genetic contribution to watercore and mealiness in the ‘Fuji’-related apples was demonstrated by Kunihisa et al. [10], who examined genomic dissection of ‘Fuji’ using 115 accessions of its descendants and parents. In that study, one quantitative trait loci (QTL) was detected for the following traits: degree of watercore and mealiness, acidity, and harvest day. The QTL for a high degree of watercore was detected in the middle of chromosome (chr)14, whereas the one for mealiness was detected at the middle of chr1. ‘Fuji’ has inherited haplotypes from both ‘Delicious’ and ’Ralls Janet’. The haplotype of ‘Fuji’ derived from ‘Delicious’ in the chr14 region dominantly causes watercore, whereas one in the chr1 region causes mealiness. For mealiness, another QTL associated with *MdPG1* was detected from different F_1_ population [40]. So far, as ‘Fuji’ descendant, however, 90% of selected cultivars or superior breeding lines have inherited the haplotype of ‘Fuji’-derived ’Ralls Janet’ at the region of chr1 [10,48].

Sadamori, a leader of the ‘Fuji’ breeding team, recounted that most of the seedlings of ‘Ralls Janet’ and ‘Delicious’ generated sweet but mealy fruit in his memoir [49]. Among them (592 fruits), they found only two crisp and nonmealy fruits. One of them, which exhibited excellent flavor, was what eventually became ‘Fuji’ [49]. There was only a 0.3% frequency of nonmealy flesh from that cross; however, nonmealy phenotypes are more common in ‘Fuji’-related accessions. Therefore, newly developed watercore-susceptible lines derived from ‘Fuji’ have an improved chance of possessing both excellent flavor and texture. Additional genetic information on the turgor reduction after harvest and its physiological understandings will help further improve and maintain the crispness of apples.

### 3.2. Watercore and Texture

Juiciness positively contributes to perceived freshness and is dependent on water content [50]. The water content of watercored apples is higher than that of nonwatercored apples, which is caused by the fluid within intercellular spaces or apoplast that causes watercore. Iwanami et al. [50] reported that both the water content of the whole fruit and apoplast tissues positively correlated with juiciness, affirming that watercored apples exhibit greater juiciness than nonwatercored apples. Although the report did not refer to watercore, the juiciest apple, ’Oyume’, in their data is a cultivar that generally develops rich watercore. Maintaining the perception of freshness in apples, which are commonly stored for relatively long periods of time, is crucial for continued consumer appeal and requires appropriate storage conditions.

**Figure 4:**
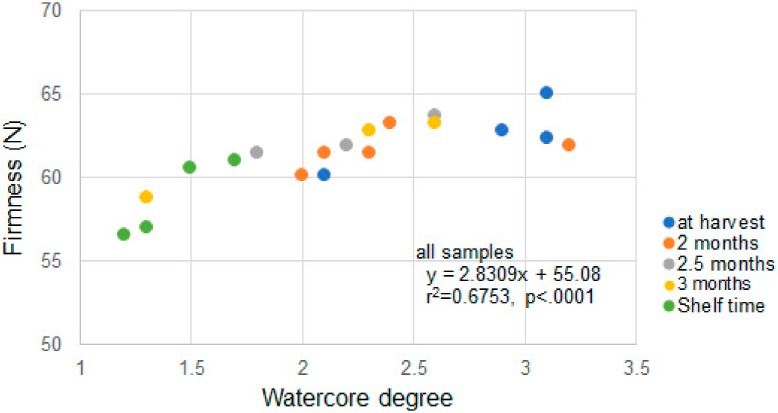
Watercore degree (0–4) and flesh firmness of ‘Fuji’ apple. Reproduced with permission from Onodera [51].

In order to establish a storage technique for high-quality watercored ‘Fuji’, Onodera et al. (2010) [51] investigated storability of apples that exhibited >30% watercoring. Time-course measurements of firmness and watercore degree were taken during 3 months of storage and 14 days of shelf time under regular atmosphere (RA). Both watercore degree and firmness decreased with time, and these exhibited significant positive correlation to each other (Figure 4). These results were in agreement with those of a previous report of Bowen and Watkins [14], which stated that flesh firmness at harvest initially tended to decrease with watercore scores and then significantly increased as watercore enhanced. These data suggest that highly watercored apples may maintain a firmer texture than lesser watercored apples for a few months after harvest. Further case examples are required.

Being a major plant growth regulator and ripening hormone, ethylene is considered to be involved in the softening of apples [41,52]. Internal ethylene concentration (IEC) was measured in relation to watercore degree. Bowen and Watkins [13] reported that IEC increased with the watercore degree, whereas Argenta et al. [53] reported that IEC was higher in fruit with a low watercore score, and it decreased in fruits with a high watercore score compared with watercore-free. There have been few studies regarding the firmness of watercored apples under storage and its regulation, limiting what is currently known. As watercored apples gain popularity, further studies will likely greatly elucidate the relationship between firmness and watercoring.

## 4. Watercore During Storage

Watercored apples are likely to develop physiological disorders in the flesh, including watercore breakdown, internal browning, and various other disorders and, in some cases, worsen the degree of existing disorders [3,4,5,53,54,55,56]. These disorders often hinder the storability of apples and their use as a long-shelf life commodity. Watercore development is accompanied by photosynthetic carbohydrate accumulation in the fruit; consequently, as harvest is delayed, the degree of watercore increases [14,57,58]. Therefore, watercore-susceptible cultivars are often harvested long before maturity at the expense of sweet flavor.

**Figure 5:**
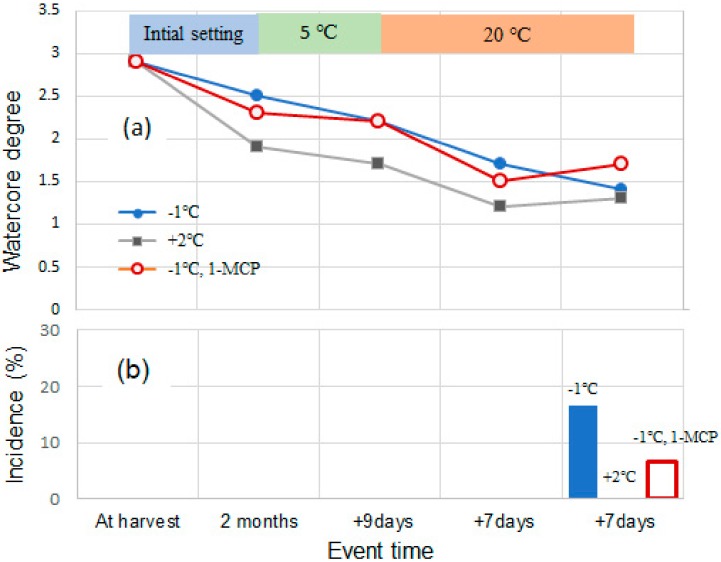
Time course of watercore degree (0–4) and watercore breakdown incidence (area %) in ‘Fuji’ apple. *n* = 30. (**a**) Watercore degree, (**b**) watercore breakdown incidence. Watercore breakdown was not observed in the apples that were initially stored at 2 °C. Reproduced with permission from Onodera [51].

Onodera et al. [51] investigated the storability of highly watercored ‘Fuji’ with or without 1-methylcyclopropene (1-MCP) treatment for 3 months. Watercore breakdown did not occur until 3 months after harvest, irrespective of 1-MCP treatment and temperature settings. Another experiment in Figure 5 presents a time course of watercore degree and incidence of watercore breakdown during shelf life. The storage conditions were set at −1 °C or 2 °C for an initial 2 months and at 5 °C for 9 days followed by 20 °C for 14 days. Watercore degrees gradually decreased in all treatments during the experiment, and the incidence of watercore breakdown was detected at 14 days after storage at 20 °C. These results indicate that watercored ‘Fuji’ can be stored for up to 3 months under RA with refrigeration.

**Figure 6:**
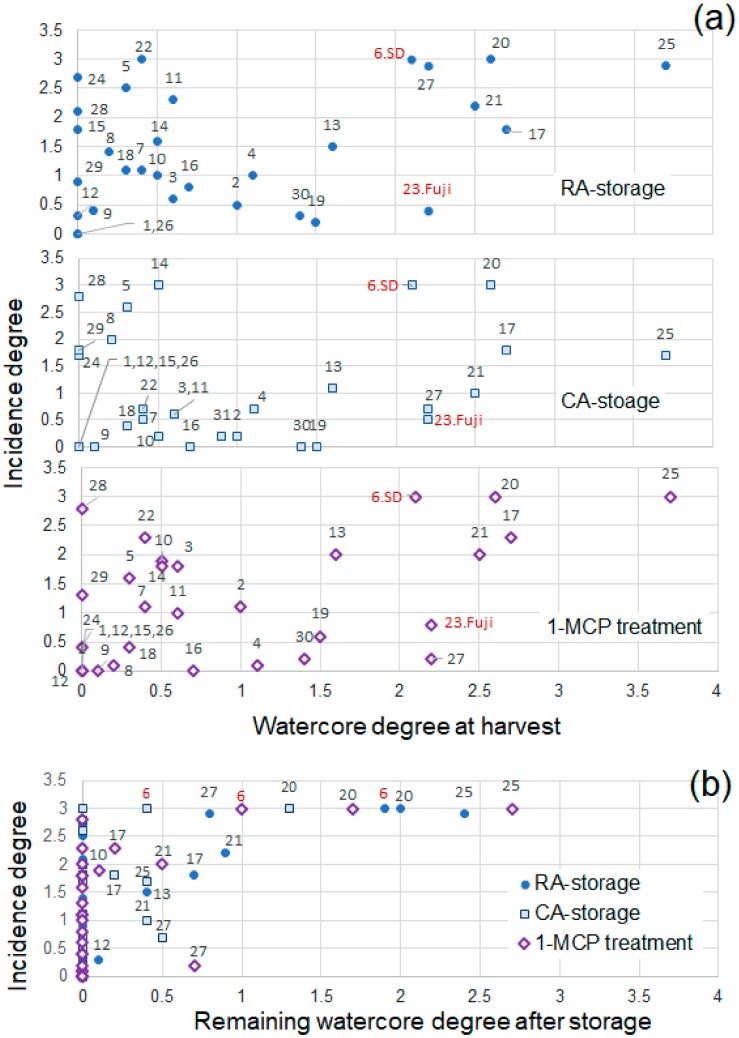
Watercore degree (0–4) and incidence of flesh browning disorders (0–3) in various apple cultivars. (**a**) Incidence degree to watercore degree at harvest; (**b**) Incidence degree to watercore degree after storage. *n* = 10. 1: ‘Shuyo’; 2: ‘Seimei’; 3: ‘Shinano Sweet’; 4: ‘Sekaiichi’; 5: ‘Morinokagayaki’; 6: ‘**Starking Delicious’ (SD)**; 7: ‘Kitarou’; 8: ‘Jona Gold’; 9: ‘Koutaro’; 10: ‘Yoko’; 11: ‘Megumi’; 12: ‘Aori 27′; 13: ‘Aikanokaori’; 14: ‘Mutsu’; 15: ‘Shinano Gold’; 16: ‘Mahe 7′; 17: ‘Hokuto’; 18: ‘Orin’; 19: ‘Aori 15′; 20: ‘Gunma Meigetsu’; 21: ‘Koukou’; 22: ‘Slim Red’; **23: ‘Fuji’**; 24: ’Mellow’; 25: ‘Koutoku’; 26: ‘Ambitious’; 27: ‘Romu 50′; 28: ‘Granny Smith’; 29: ‘Cripps Pink’; 30 ‘Aori 21′; 31: ‘Fuji’ (bagged). Reproduced with permission from Kasai et al. [59].

Storage performance over a much longer duration than that reported by Onodera et al. [51] was reported by Kasai et al. [59] to determine which cultivars were resistant to physiological disorders and deterioration of flavor and texture. Apples of 30 cultivars were harvested at their commercial harvest time in the fall and stored under RA, CA, and 1-MCP treatment until mid-June of the next year at 0 °C followed by under RA at 20 °C for 5 days. The watercore degree and flesh browning disorder, which is regarded as a serious problem in watercored apples, were analyzed, and flesh browning disorder was found to occur in most cultivars irrespective of the presence or absence of watercore at harvest, except for ’Shuyo’ and ’Ambitious’. Figure 6a presents the relationship between watercore scores at harvest and flesh browning disorder incidence after storage. Seven cultivars scored >2 in watercore, and most of them had high incidence of physiological disorders. However, the incidence in ‘Fuji’ was low for the score of watercore, which may have been due to the disappearance of watercore. The remaining watercore after storage and the incidence of flesh browning disorders are presented in Figure 6b. Flesh browning occurred irrespective of the remaining watercore score and storage condition; however, highly watercored remaining fruits exhibited severe flesh browning without exception. Watercore is not the only cause of the flesh browning disorder; however, prolonged, severe watercore greatly enhances the severe incidence in flesh.

Storage longer than 4–5 months usually utilizes several treatments to suppress respiration and ethylene function, which results in an inhibition of aroma synthesis. This inhibits the generation of distinct, sweet aroma, which is the advantage of fresh watercore-rich apples and which cannot be produced after CA storage. In other words, watercored apples should be eaten within a few months of harvest or earlier, especially highly watercore-rich fruits.

Based on work from several previous studies, watercore development can be enhanced or inhibited using cultural techniques on watercore-susceptible cultivars. Watercore is promoted by low or high air temperatures during the preharvest period, large fruit, poor calcium concentration, high nitrogen and boron nutrition, a high leaf-to-fruit ratio, excessive fruit thinning, high or low light exposure, growth in volcanic ash soil, ethrel (ethephon) and gibberellin treatment, and girdling of the trunk and limbs [9]. Therefore, to develop rich watercore for a premium product, fruits are allowed to increase photosynthate accumulation by means of increasing the light received by the leaves and fruits and harvesting at full maturity. To maintain a long shelf life without the watercore physiological disorder, photosynthate accumulation in fruits is limited by earlier harvesting and fruit bagging. Apple producers choose one of these cultivation methods according to demand and their business policies.

## 5. Conclusions

Watercore in apple had been avoided for years due to the mealy texture and brown flesh incidence associated with it. Currently, however, watercore-rich apples are gaining popularity, mainly in Asian countries. ‘Fuji’, the first rich-watercored cultivar that is free from texture deterioration, greatly contributed to the paradigm shift. ‘Fuji’ resulted from a cross made in 1939, and though many decades have passed, the potential of ‘Fuji’ as a high-quality apple is still being shown by integration of diverse analytical methods, such as instrumental analysis and sensory, chemical, physiological, and genetic aspects. Still, there are many unresolved issues related to apple quality. Expanding the understanding of the nature and physiology of apple will continue to lead to improvements in apple quality by utilizing various concepts, approaches, and techniques.

## Figures and Tables

**Table 1:** Sensory evaluation for watercored and nonwatercored ‘Fuji’ apples.

Sample Status	Overall Acceptability	Overall Intensity		Sensory Attribute				
		Aroma	Taste	Green	Fruity	Floral	Sweet	Sour
Nonwatercored	3.0	4.0	4.1	4.3	4.0	3.1	3.9	4.0
Watercored	3.5	4.5	4.2	3.6	4.4	4.2	4.6	3.2
Significance	**	**	ns	**	*	***	**	***

Apple: Products of a commercial orchard, peeled, cored, and cut into bite-size pieces just before being served. Evaluation: a seven-point categorical scale (1–7); 29 panelists trained for quantitative destructive analysis, 10 females and 19 males. Taste was evaluated with nose clip to eliminate the influence of aroma. Significance: *, **, *** indicate significant differences at the level of *p* < 0.05, *p* < 0.01, or *p* < 0.001, respectively, using paired *t*-test; ns means not significant. Reproduced with permission from Tanaka et al. [13].

**Table 2:** Sugar profiles and estimated perceived sweetness of various apple cultivars.

Cultivar	Watercore	Sugar Contents (g/100 g FW)				Estimated Sweetness		Ref.
		Fructose	Glucose	Sucrose	Sorbitol	(a)	(b)	
‘Fuji’	absent	5.7	3.1	1.5	0.4	12.0	12.3	[14] ^1^
	present (richest level)	5.5	2.2	3.4	1.2	12.7	13.2	
‘Fuji’	absent	6.6	2.3	1.8	0.5	12.3	13.4	[15]
	present	5.6	1.9	1.8	1.5	11.3	11.7	
‘Gloster’	absent	5.2	1.9	3.2	0.3	11.5	12.4	
	present	3.8	1.7	2.6	0.9	9.2	9.6	
‘Delicious’	absent	6.4	1.7	2.2	0.2	11.9	13.1	
	present	5.1	1.5	2.0	1.0	10.2	10.7	
‘Esperiega’	absent	7.2	1.51	2.8	0.8	13.7	14.7	[16]
	present (nonaffected site)	6.9	2.0	3.2	1.5	14.4	15.0	
	present (affected site)	6.3	2.2	1.5	2.9	13.0	12.6	
‘Winesap’	absent	3.2	4.0	3.8	0.9	11.3	11.6	[17] ^1^
	present (nonaffected site)	3.4	4.2	4.1	1.3	12.2	12.4	
	present (affected site)	3.0	3.7	3.8	1.8	11.4	11.1	

^1^ Original sugar contents were converted to g/100 g FW. Estimated sweetness: (a) (1.0 [sucrose]) + (1.3 [fructose]) + (0.7 [glucose]) + (0.7 [sorbitol]) [22]; (b) = (1.0 [sucrose]) + (1.5 [fructose]) + (0.76 [glucose]) [23].

## Supplementary Materials

The following are available online. Table S1: Intensity of volatiles in watercored and nonwatercored ‘Fuji’ and ‘Koutoku’ apples, Table S2: Intensity of water-soluble compounds in watercored and nonwatercored ‘Fuji’ and ‘Koutoku’ apples.
