# Supplementary material for: Flavor and Texture Characteristics of ‘Fuji’ and Related Apple (Malus domestica L.) Cultivars, Focusing on the Rich Watercore
Source: Molecules. 2020 Mar 2;25(5):1114. doi: 10.3390/molecules25051114 (PMC7179182; doi:10.3390/molecules25051114)
Supplement: Supplementary file 1 [file molecules-25-01114-s001.zip › Table S1 Intensity of volatiles in watercored and nonwatercored 'Fuji' and 'Koutoku' apples.pdf]

Intensity of volatiles in watercored and nonwatercored 'Fuji' and 'Koutoku' apples

Volatiles	'Fuji'		'Koutoku'		Signifficance		
	WC	nonWC	WC	nonWC	cultivar	WC	interaction
Alcohols							
Methanol	1	1.95	0.45	0.48	***	**	*
iso-Propanol	1	0.07	0.00	0.00	***	***	***
Ethanol	1	2.42	1.77	2.08	ns	*	ns
Propanol	1	0.48	1.60	0.18	ns	*	ns
2-Methylpropanol	1	0.92	0.99	0.42	ns	*	ns
Butanol	1	0.72	0.51	0.12	***	***	ns
2-Methybutanol	1	0.69	0.49	0.26	**	**	ns
Pentanol	1	0.81	0.21	0.12	***	*	ns
Hexanol	1	0.69	0.28	0.13	***	*	ns
Octanol	1	1.35	0.71	0.16	ns	ns	ns
Acetol	1	0.50	7.37	5.06	ns	ns	ns
(E) -2-hexen-1-ol	1	0.69	0.00	0.04	***	ns	ns
6-Methyl-5-hepten-2-ol	1	20.33	12.10	6.19	ns	ns	ns
2-Ethylhexanol	1	0.91	0.61	0.91	ns	ns	ns
Furfuryl alcohol	-	-	5[§]	5[§]	*	ns	ns
(E)- Linalool oxide	1	1.30	-	-	**	ns	ns
Aldehydes							
Butanal	1	0.42	0.48	0.05	***	***	*
2-Methylbutanal	1	-	-	-	***	***	***
Petanal	1	0.58	-	-	***	***	***
Hexanal	1	0.63	0.07	0.06	***	***	***
Hepanal	1	0.42	-	-	***	**	**
Octanal	1	0.21	-	-	**	*	*
Nonanal	1	0.52	0.00	0.27	***	ns	**
Decanal	1	0.27	0.56	0.81	ns	ns	ns
(E) 2-Hexenal	1	0.69	0.11	0.13	***	ns	*
(E) 2-Heptenal	1	0.75	0.01	0.04	***	*	**
(E) -2-Octenal	1	0.56	0.01	0.03	***	**	**
2,4-Heptadienal	1	1.45	-	-	***	**	**
Benzaldehyde	1	2.54	0.44	0.20	**	ns	ns
Ketones							
acetone	1	0.65	0.42	0.30	***	**	ns
1-Octen-3-one	1	0.35	-	-	***	*	*
6-methyl-5-hepten-2-one	1	0.65	0.15	0.15	***	ns	ns
Acids							
Acetic acid	1	9.02	3.41	4.93	ns	ns	ns
2-Methylbutanoic acid	-	-	5[§]	4[§]	ns	ns	ns
Esters							
Methyl acetate	1	75.38	0.32	3.52	***	***	***
Methyl propanoate	1	17.55	-	-	***	***	***
Methyl butanoate	-	6[§]	-	-	**	**	**
Methyl 2-methylbutanotae	-	6[§]	4[§]	5[§]	***	***	***
Methyl hexanoate	1	6.26	2.67	0.40	ns	ns	ns
Ethyl acetate	1	156.07	2.07	28.74	***	***	***
Ethyl propanoate	1	165.34	7.96	125.16	ns	***	***
Ethyl iso-butanoate	1	503.41	0.00	0.00	**	**	**
Ethyl butanoate	1	30.44	1.34	10.70	**	***	**
Ethyl 2-methylbutanoate	1	87.05	1.22	30.29	*	**	*
Ethyl pentanoate	1	0.61	-	1.74	ns	ns	ns
Ethyl haxanoate	-	7[§]	-	6[§]	***	***	***
Ethyl tiglate	-	5[§]	-	-	***	***	***
Ethyl octanoate	-	-	3[§]	4[§]	ns	ns	ns
Ethyl 3-hydroxyhexanoate	1	94.31	18.28	220.94	ns	*	ns
Prppyl acetate	1	0.56	2.09	0.36	ns	**	*
Propyl propanoate	1	0.53	1.31	0.31	ns	ns	ns
Propyl 2-methylbutanoate	1	0.26	0.23	0.07	ns	ns	ns
Propyl hexanoate	1	0.31	1.04	0.12	ns	*	ns
Butyl acetate	1	0.48	0.16	0.02	***	**	ns
Butyl propanoate	1	0.17	-	-	***	**	**
Butyl butanoate	1	0.21	-	-	***	***	***
Butyl 2-methylbutanoate	1	0.24	0.05	0.00	**	**	*
Butyl hexanoate	1	0.39	0.74	0.05	ns	*	ns
Butyl octanoate	1	0.33	-	-	**	ns	ns
iso-Butyl acetate	1	0.35	0.15	0.00	**	*	ns
iso-Butyl butanoate	1	0.13	0.13	0.02	***	***	**
2-Methylbutyl acetate	1	0.54	0.14	0.03	**	*	ns
2-Methylbutyl butanoate	1	4.38	-	-	ns	ns	ns
2-Methylbutyl 2-metylbutano:	1	0.46	-	-	*	ns	ns
2-Methylbutyl hexanoate	1	1.05	1.71	0.10	ns	ns	ns
Pentyl acetate	1	0.31	0.07	0.01	***	***	***
Pentyl butanoate	1	0.15	0.73	0.09	ns	**	ns
Hexyl acetate	1	0.26	0.18	0.01	***	**	***

Hexyl propanoate	-	-	5[§]	5[§]	***	**	**
Hexy butyrate	1	0.12	0.00	0.00	***	***	***
Hexyl 2-methylbutanoate	1	0.19	0.44	0.05	**	***	*
Hexyl hexanoate	-	-	5[§]	5[§]	ns	ns	ns
(E) -2-Hexenyl acetate	1	0.20	-	-	**	*	*
Others							
Toluene	1	0.63	-	-	**	ns	ns
(E,E) -α-farnesene	1	0.21	-	-	ns	ns	ns
unknown	1	0.26	-	-	***	***	***

Intensity of each volatile in non-watercored 'Fuji' was set as 1,

§; peak area in the logarithm intensity of volatile that was not detected in nonwatercore 'Fuji''

ns: not significant; *, ** and ***; significant at $p < 0.05$, 0.01, and 0.001, respectively.

WC: watercored; nonWC: nonwatercored
