# Supplementary material for: Flavor and Texture Characteristics of ‘Fuji’ and Related Apple (Malus domestica L.) Cultivars, Focusing on the Rich Watercore
Source: Molecules. 2020 Mar 2;25(5):1114. doi: 10.3390/molecules25051114 (PMC7179182; doi:10.3390/molecules25051114)
Supplement: Supplementary file 1 [file molecules-25-01114-s001.zip › Table S2 Intensity of water-soluble compounds in watercored and nonwatercored 'Fuji' and 'Koutoku' apples.pdf]

Intensity of water-soluble compounds in watercored and nonwatercored 'Fuji' and 'Koutoku' apples

water-soluble compounds	'Fuji'		'Koutoku'		Signifficance		
	WC	nonWC	WC	nonWC	cultivar	WC	interaction
Amino acids, polyamine							
L-Alanine	1	1.39	0.68	3.16	**	***	**
L-Valine	1	0.73	0.85	1.42	ns	ns	*
L-Serine	1	0.23	1.27	1.53	*	ns	ns
b-Alanine	1	0.92	1.97	4.35	*	ns	ns
L-Aspartic	1	0.02	1.78	1.12	ns	ns	ns
4-Aminobutyric	1	0.83	0.37	0.52	ns	ns	ns
L-Glutamic	1	0.61	1.91	3.52	***	ns	*
L-Asparagine	1	0.09	2.14	1.34	ns	ns	ns
Pyroglutamic	1	0.97	1.72	3.14	**	*	*
Putrescine	1	1.69	1.68	3.10	*	*	ns
Acids							
Malic acid	1	1.05	0.58	0.70	***	ns	ns
Erythronic acid	1	1.81	0.72	1.12	**	**	ns
Threonic acid	1	1.33	0.75	0.92	ns	ns	ns
Ribonic acid	1	3.86	0.77	1.21	ns	ns	ns
Shikimic acid	1	0.76	1.44	1.60	ns	ns	ns
Citric acid	1	0.73	1.12	1.14	ns	ns	ns
Dehydroascorbic acid	1	1.87	1.02	1.93	ns	**	ns
D(-)-Quinic acid	1	0.72	0.90	1.28	ns	ns	ns
Phosphoric acid	1	0.76	0.65	0.59	ns	ns	ns
Galactaric acid	1	2.14	0.99	1.75	ns	**	ns
Sugars							
Fructose	1	1.02	0.78	0.83	***	ns	ns
Glucose	1	0.81	0.68	0.64	**	*	ns
Sucrose	1	1.13	0.87	1.01	*	*	ns
Xylose	1	0.59	1.23	1.11	*	ns	ns
Arabinose	1	0.92	0.95	1.09	ns	ns	ns
Ribose	1	0.90	1.68	1.40	***	*	ns
Rhamnose	1	0.88	0.93	0.96	ns	ns	ns
Raffinose	1	1.07	1.44	1.21	**	ns	ns
Sugar alcohols							
Erythritol	1	1.12	1.90	1.73	**	ns	ns
Xylitol	1	1.21	1.00	1.07	ns	ns	ns
Sorbitol	1	2.16	0.98	1.77	ns	**	ns
Threitol	1	0.86	1.09	1.15	ns	ns	ns
myo-Inositol	1	1.03	1.68	1.90	**	ns	ns

Intensity of each water-soluble compound in nonwatercored 'Fuji' was set as 1
ns: not significant; *, ** and ***; significant at $p < 0.05$, 0.01, and 0.001, respectively.
WC: watercored; nonWC: nonwatercored
